# Herpesvirus Diseases in Humans and Animals: Recent Developments, Challenges, and Charting Future Paths

**DOI:** 10.3390/pathogens12121422

**Published:** 2023-12-07

**Authors:** Miroslava Šudomová, Sherif T. S. Hassan

**Affiliations:** 1 Museum of Literature in Moravia, Klášter 1, 664 61 Rajhrad, Czech Republic; 2 Department of Applied Ecology, Faculty of Environmental Sciences, Czech University of Life Sciences Prague, Kamýcká 129, 165 00 Prague, Czech Republic; sherif.hassan@seznam.cz

## 1. Introduction

Herpesviruses, a family of enveloped DNA viruses, pose significant threats to both humans and animals. They cause a spectrum of diseases, ranging from mild, self-limiting conditions to severe, life-threatening illnesses. With the ability to establish lifelong relationships with their hosts and trigger recurrent outbreaks, herpesviruses have emerged as formidable players in the realm of infectious diseases [1,2,3]. Human herpesviruses, including herpes simplex virus (HSV), varicella-zoster virus (VZV), cytomegalovirus (CMV), Epstein–Barr virus (EBV), Kaposi’s sarcoma-associated herpesvirus (KSHV), and others, continue to exert a substantial impact on global public health [4,5]. Likewise, the animal kingdom encounters viral obstacles, as equine, bovine herpesviruses, feline, and canine herpesvirus, pseudorabies virus, and other related viruses cause considerable risks to agriculture and veterinary fields [6,7,8,9].

The overlap between human and animal health features the interconnected challenges presented by these viruses [10].

## 2. Recent Developments

In recent years, significant strides have been made in elucidating the molecular biology and pathogenesis of human and animal herpesviruses. Advances in genomics, proteomics, and imaging techniques have provided unprecedented insights into the intricate interactions between these viruses and their hosts [1,11]. Furthermore, the identification of viral and host factors influencing the course of infection has opened new avenues for therapeutic interventions [12]. Cutting-edge technologies, from CRISPR-based genome editing to high-throughput sequencing, have empowered scientists to dissect the viral genome, identify novel therapeutic targets, and explore innovative vaccine strategies [13,14,15,16,17]. The development of antiviral drugs, such as acyclovir and its derivatives, has improved patient outcomes. However, concerns about drug resistance and the persistence of latent infections remain [18,19].

Overall, the integration of these diverse approaches has expanded our understanding of herpesviruses and paved the way for more effective strategies in combating these infections.

## 3. Challenges and Innovative Solutions

Navigating the complex landscape of herpesvirus research reveals a set of challenges that span various facets of virology and medicine. One prominent challenge lies in drug resistance, where, despite the effectiveness of antiviral drugs such as acyclovir, the emergence of drug-resistant strains poses a substantial threat. Ongoing surveillance, and the development of alternative therapeutic strategies, are imperative for addressing this issue [4,20,21].

The challenge of latency and reactivation in herpesvirus infections, characterized by the establishment of latent infections and periodic reactivation, adds complexity to the treatment and prevention efforts. Understanding and targeting latent reservoirs is crucial for curbing recurrent infections, with innovative solutions such as therapies targeting latent reservoirs, gene-editing technologies, and an enhanced understanding of triggers for viral reactivation. Moreover, investigating immunomodulatory agents to enhance immune surveillance shows promise [22,23,24,25]. Furthermore, a possible link between herpesvirus reactivation and various conditions, including long COVID-19 and chronic fatigue syndrome, has been suggested. The stress on the immune system during acute COVID-19 may contribute to reactivation and, in chronic fatigue syndrome, viral reactivation could impact immune regulation and chronic inflammation. Ongoing research aims to establish clearer connections and potential therapeutic approaches for these conditions [26,27,28].

In the dynamic field of vaccine development, despite notable research progress, crafting a safe and effective herpesvirus vaccine proves to be an ongoing challenge. The complex evasion tactics employed by these viruses necessitate innovative strategies to elicit robust and enduring immune responses. Advancements in mRNA vaccine technologies, the identification of conserved viral targets for vaccine development, and the exploration of novel adjuvants are all at the forefront of efforts to conquer this challenge [29,30,31,32,33]. Moderna’s exploration of RNA-based vaccines against EBV and CMV aligns with these innovative approaches, offering potential breakthroughs in the ongoing battle against persistent viral infections [34].

Severe neurological complications, including HSV-induced encephalitis, present another challenge for herpesvirus research. Unraveling the mechanisms behind viral invasion of the nervous system is essential for preventing and treating these complications. Innovative solutions include developing antiviral drugs with enhanced blood–brain barrier penetration, exploring neuroprotective agents, and understanding the host factors influencing neurological outcomes [35,36,37,38,39].

The association of certain herpesviruses with an increased risk of cancers adds a layer of complexity to the research landscape. Innovative strategies targeting viral oncogenes, immuno-therapies for cancer prevention, and the advancement of understanding viral-induced cellular changes are key solutions to address this challenge [40,41,42].

Furthermore, the interplay between human and animal health, exemplified by simian herpesviruses, emphasizes the intricate link between them. Predicting and preventing cross-species transmission events requires a holistic One Health Approach. Strengthening surveillance at the human–animal interface, promoting interdisciplinary collaborations, and developing targeted interventions emerge as innovative solutions to break the chain of transmission between species [10,43,44].

## 4. Charting Future Paths

As we delve into the complex realm of herpesvirus diseases in humans and animals, envisioning future frontiers becomes pivotal in shaping effective strategies for their prevention, treatment, and overall management. The convergence of cutting-edge technologies, such as advanced genomics and targeted antiviral therapies, promises to revolutionize our approach. Personalized medicine tailored to individual immune responses, coupled with the development of broad-spectrum antivirals, stands out as a potential frontier. Moreover, the integration of artificial intelligence in predictive modeling and vaccine design holds potential for staying ahead of the evolving herpesvirus strains. Collaborative, interdisciplinary efforts spanning virology, immunology, and computational sciences will be essential in navigating these frontiers. As we chart a course forward, a holistic understanding that transcends species boundaries will be key, fostering a future where innovative solutions ensure the effective control and mitigation of herpesvirus diseases in both human and animal populations.
